# Horizontal transfer of matrix metalloproteinase genes links early animal and microbial evolution

**DOI:** 10.1186/s13062-025-00700-4

**Published:** 2025-11-05

**Authors:** Chris Parsons, Gregory P. Fournier

**Affiliations:** 1 Department of Earth, Atmospheric and Planetary Sciences, Massachusetts Institute of Technology, Cambridge, MA USA; 2 Department of Geosciences, Auburn University, Auburn, AL USA

**Keywords:** Horizontal gene transfer, Molecular dating, Phylogeny, Relative dating, Metazoa, Phanerozoic, MMPs, Collagen

## Abstract

**Background:**

The early evolution of animals is characterized by the emergence of complex tissues, organs, and integument, made possible in part by the diversification of groups of structural proteins. The abundance of this new kind of organic material in the environment would have provided novel nutrient opportunities for microbes, as part of the beginnings of animal-microbial coevolution. Indeed, a diverse ensemble of extant microbial groups appear to possess the enzymatic ability to cleave collagen, the most abundant animal-specific protein, through the use of matrix metalloproteinases (MMPs). In animals, MMPs serve to reshape the extracellular matrix in the course of development, but their prevalence in the microbial world has been largely overlooked.

**Results:**

MMPs have extensive diversity in Bacteria, Eumetazoa, and Streptophyta. We show that in marine metagenomes, MMP abundance is highly correlated with chitinase abundance, implying that even microbial MMPs are associated with animal-derived substrates. Reconstructing the phylogeny of MMP proteins reveals a history of rapid diversification, as well as multiple interkingdom and interdomain horizontal gene transfers. Included among these is a transfer to the ancestral lineage of the archaeal family Methanosarcinaceae, constraining this group to postdate the evolution of collagen, and therefore animal diversification.

**Conclusions:**

MMPs have an unusual genetic history, marked by multiple instances of gene transfer between bacteria and multicellular eukaryotes, a smoking gun for some of the earliest coevolution between prokaryotes and metazoans. By calculating an end-Permian divergence of *Methanosarcina*, we demonstrate that the phylogenies of substrate-specific enzymes can provide valuable older-bound age calibrations for improving molecular clock age estimates across the Tree of Life.

**Supplementary Information:**

The online version contains supplementary material available at 10.1186/s13062-025-00700-4.

## Background

The crucial innovation associated with the origin of complex animals is the evolution of tissues and organs, which greatly expanded their available phenotypic space and allowed for vastly increased morphological and ecological diversity. This series of innovations was facilitated by the evolution of proteins that support the extracellular matrix (ECM) by binding cells together and coordinating various intercellular activities [1]. While ECMs are found in all but the most basal metazoans [1], the vertebrate ECM is known to host an especially diverse suite of ECM proteins, most of which arose through duplication of pre-existing genes [2].

Collagen, probably the most well-known of the ECM proteins, is largely responsible for the structural integrity of animal tissues, while also participating in many intercellular signaling pathways [3]. It is also one of the most abundant proteins within animal bodies; collagen accounts for ~ 30% of the total mammalian protein content [4]. While 28 different families of collagens have been identified in vertebrates, only 3 have been found in all (non-placozoan) animal phyla: fibrillar collagens, basement membrane type IV collagens, and multiplexins [3, 4]. Some bacteria have been found to produce collagen as either a virulence factor or as a biofilm support, but the limited taxonomic distribution of these proteins indicates that they probably do not represent an independent evolutionary origin, and likely arose due to relatively recent horizontal gene transfer (HGT) from animals [5]. Therefore, as collagen apparently originated within Metazoa, the origin of and diversification of animals coincided with the introduction of large quantities of this new substance into the environment—a resource that microbes would rapidly evolve to exploit.

**Fig. 1:**
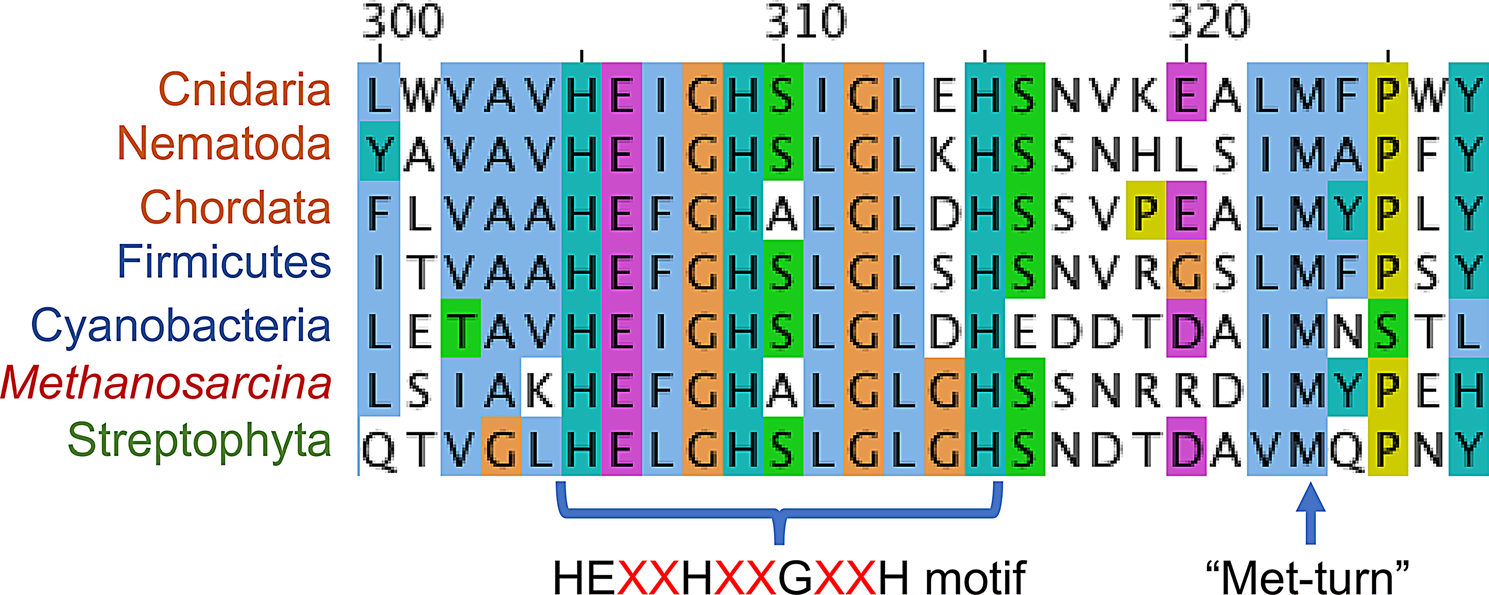
Alignment of MMP active sites in seven distantly-related taxa. Residues are colored based on the Clustal X coloring scheme. From top to bottom, the sequences correspond to: *Hydra vulgaris* (NP_001296676.1), *Caenorhabditis elegans* (NP_001333542.1), *Mus musculus* (AAX90605.1), *Bacillus cereus* (WP_102956764.1), *Phormidesmis priestleyi* (KPQ35123.1), *Methanosarcina barkeri* (AKB83795.1), and *Glycine max* (XP_014626951.1)

Essential to the function of the ECM and development of an animal body over time is the ability to remodel and break down structural ECM components. This is achieved through the use of matrix metalloproteinases (MMPs), also known as matrixins, which are able to cleave one or more ECM proteins, allowing for crucial functions such as growth and healing [6]. MMPs belong to the broader superfamily of zinc-peptidases, also known as zincins or clan MA [7]. Clan MA proteins are highly divergent, and share very low (generally < 20%) sequence similarity to one another [8], causing a great deal of uncertainty regarding their higher order evolutionary relationships. All MMPs contain a catalytic zinc ion in the active site, as well as a structural zinc ion and a structural calcium ion [6]. Within the broader diversity of zinc-peptidases, MMPs are united both phylogenetically, and by a consensus sequence at the catalytic zinc-binding site: HEXXHXXGXXH [9] (Fig. 1).

Genes encoding MMPs have been duplicated many times within vertebrates, while invertebrate genomes generally encode a more reduced suite of MMPs [6, 8]. The taxonomic distribution of MMPs within Metazoa remains unclear, with some studies reporting their presence in all major clades of animals [1] and others reporting their absence in Ctenophora, Porifera, and Placozoa [8]. Therefore, uncertainty remains as to whether MMPs initially evolved in the metazoan ancestor lineage, or following the divergence of Ctenophora and Porifera (and possibly Placozoa depending on its phylogenetic placement).

MMPs have additionally been identified in both land plants and bacteria, and these variants share high sequence similarity to animal MMPs [8]. A previous study has proposed that bacterial MMPs are mostly found within gut microbiomes, and therefore likely were initially transferred into bacteria in gut environments [8]. However, to date, no studies have experimentally tested the ability of bacterial MMPs to cleave collagen or other animal ECM proteins. Plant MMPs have been shown to degrade both animal and plant matrix proteins, hinting at their role as mediators of the plant ECM, despite the different assemblage of ECM proteins in plants [10]. Previous studies have also noted the tendency of invertebrate and plant MMPs to group together in phylogenetic analyses [6, 8].

The taxonomic distribution of MMP proteins within both eukaryote and microbial groups suggests that an improved phylogenetic reconstruction of their history could provide a novel means of establishing divergence times within microbial lineages. The ages of most major microbial groups are almost completely unconstrained by absolute dating from the fossil record, aside for a few groups (e.g., Cyanobacteria), whereas otherwise the record remains sparse and often ambiguous [11]. Lipid biomarker records are potentially useful for dating only a subset of microbial groups (e.g., [12–14]). Geochemical records such as microbially mediated isotopic fractionations (e.g., [15–17]) may potentially provide younger-bound constraints on the antiquity of different energy metabolisms, but not specific clades of microbes, as genes for these metabolisms have been transferred between multiple disparate groups (e.g., [18]). Therefore, indirect means of dating become essential, constraining microbial divergence times via connections to groups of organisms with a better-established fossil record, especially crown eukaryotes such as metazoans, fungi, algae, and land plants. This can be accomplished by observing the co-evolution of host-symbiont systems (e.g., [19, 20]), using horizontal gene transfer to propagate fossil constraints (e.g., [21–23]), through inferences of substrate utilization (e.g., [24]), or some combination thereof (e.g., [25]).

The transfer of MMP genes between metazoan and microbial groups is potentially especially useful in propagating such absolute age constraints, as collagen originated along the stem lineage of Metazoa, estimated by fossil-calibrated molecular clock studies as potentially spanning from 920 Ma [26] to 650 Ma [27]. Furthermore, the antiquity of collagen means this constraint is potentially older than those provided by other eukaryote-specific substrates, such as chitin or lignin, and therefore may have utility in dating microbial lineages of higher taxonomic rank. Combined with molecular clock analyses, these inferences can be highly informative for establishing a higher-resolution timeline of microbial evolution, especially during the Late Neoproterozoic and Phanerozoic, a history that would otherwise remain elusive.

Here, we provide metagenomic contextual evidence that bacterial MMPs degrade animal collagens by observing significant co-occurrence with bacterial chitinase genes, and show that an expanded phylogeny of MMP proteins reveals a likely origin within Bacteria. We infer that the diversification of bacterial MMPs was followed by multiple HGT events within Bacteria, as well as two interdomain transfers into stem Streptophyta and stem Parahoxozoa. Most intriguingly, we show a likely HGT to the ancestor of the archaeal methanogen group Methanosarcinaceae. Revised molecular clock analyses of Methanosarcinaceae incorporating the older-bound constraint provided by the presence of MMPs produces age estimates consistent with, and more precise than, previously published ages for Methanosarcinaceae and its descendant lineages.

## Methods

### Sequence selection and alignment

Sequences generated by HMM searches of the NCBI non-redundant protein database [28] were downloaded from Pfam (family PF00413) [29]. Redundant or duplicate sequences were removed using Jalview with a redundancy cutoff of 95% [30], as were any sequences that either did not include the canonical HEXXHXXGXXH motif in the active site or were fewer than 100 amino acids long. Sequences were then realigned using MUSCLE with default parameters [31] and an initial phylogeny was created.

### Tree construction

Phylogenies were generated using IQTree (version 1.6.3) [32]. The model was selected using ModelFinder [33], limited to the WAG, LG, and JTT substitution matrices to reduce computation time. The best-fitting model for the final phylogeny was identified as WAG, with empirical amino acid frequencies, and a FreeRate model with 10 rate categories (WAG + F + R10). To assess the confidence of particular bipartitions, 1000 ultrafast bootstrap (UFBoot) [34] replicates and 1000 Shimodaira–Hasegawa approximate likelihood-ratio test (SH-aLRT) [35] replicates were generated. To refine the tree, sequences with ambiguous placements on long branches or large poorly aligned regions were removed from the alignment. Additionally, resolution was increased in areas of interest (such as Methanosarcinaceae) by adding additional sequences through targeted BLAST searches. The final MMP phylogeny contained 1,074 sequences (Description Supplementary Material 1).

A Bayesian consensus tree of sequences belonging to family Methanosarcinaceae was constructed using Phylobayes version 4.1c (C20 mixture model, all other parameters default) [36] (Supplementary Material 2). Convergence was assessed using an automatic stopping rule for two simultaneous chains with all effective sizes exceeding 50 and all parameter variances lower than 0.2.

### Outgroup root testing

Initial attempts to root the MMP phylogeny using M12B as an outgroup led to the inference of a metazoan root (Supplementary Material 5). However, the rooting was a tip rooting and strongly implied that long branch attraction and low sequence conservation between the two families led to a poor alignment. To test if the outgroup supported a metazoan MMP rooting more than a bacterial rooting, the likelihoods of all possible rootings were calculated. A small MMP phylogeny (104 sequences) and M12B phylogeny (36 sequences) were separately calculated and then connected at all possible rootings for both families, excluding tip-rootings. Using IQTree, the individual likelihoods of the resultant set of trees were calculated and compared to the highest likelihood using an approximately unbiased likelihood test (Fig. 6).

### Molecular clock age inference

To test the effects of the application of the Methanosarcinaceae calibration on archaeal age estimates, a previously published protein alignment and associated phylogeny [23] was used. Homologous ribosomal sequences of five recently-sequenced Methanosarcinaceae members were also identified via protein BLAST and added to the dataset, with subsequent realignment and reconcatenation: *Methanosarcina siciliae*, *M. lacustris*, *M. sp. WH1*, *Methanimicrococcus blatticola*, and *Methanomethylovorans hollandica*. To determine the placements of these sequences in the phylogeny, a Bayesian consensus tree was constructed, using the same parameter settings as described in *Methods*, but including only Methanosarcinaceae and close relatives (Fig. 4b; Supplementary Material 3).

Divergence times were estimated using Phylobayes version 4.1c [36] with a C20 mixture model and a root prior of 3.9 ± 0.23 Ga, and calibrated with 1.6 Ga endolithic cyanobacterial fossils that have been attributed to family Chroococcales [37], as in the original study (Supplementary Material 4). A hard older bound of 850 Ma was additionally applied to crown Methanosarcinaceae, imposing the constraint provided by the MMP HGT; consequentially, the age of crown Methanosarcinaceae, determined by the branch rate model and likelihood of sequence data, was left free to vary between 0 and 850 Ma. Uncorrelated gamma, log-normal, and CIR branch rate models were all implemented together with both uniform and birth-death priors.

## Results

### Metagenomic correlation of bacterial MMPs with chitinases

Three major groups of collagenases are known to exist within microbial genomes: the MMP family, M9 family, and U32 family proteinases [9]. In the absence of experimental data, sequence alone cannot inform the specificity of these enzymes for animal collagen, as microbial collagen-like proteins exist (e.g., [38]). Therefore, microbial “collagenases” may have broad substrate specificity not linked to the availability of animal collagen. In order to test the likely involvement of each collagenase family in the degradation of animal-sourced material, a metagenomic chitinase-collagenase co-occurrence test was performed, using the TARA oceans dataset [39]. The original researchers provided InterProScan [40] annotations of the reads from globally-distributed ocean metagenomes of various depths, and we used their abundance values to calculate correlation coefficients for genes associated with M10 peptidases (IPR001818), U32 collagenases (IPR020988), and M9 collagenases (IPR002169), against chitinases belonging to Glycosyl Hydrolase Family 18 (IPR002169). Importantly, these alternative collagenases, M9 and U32, are almost exclusively found in Bacteria.

**Fig. 2:**
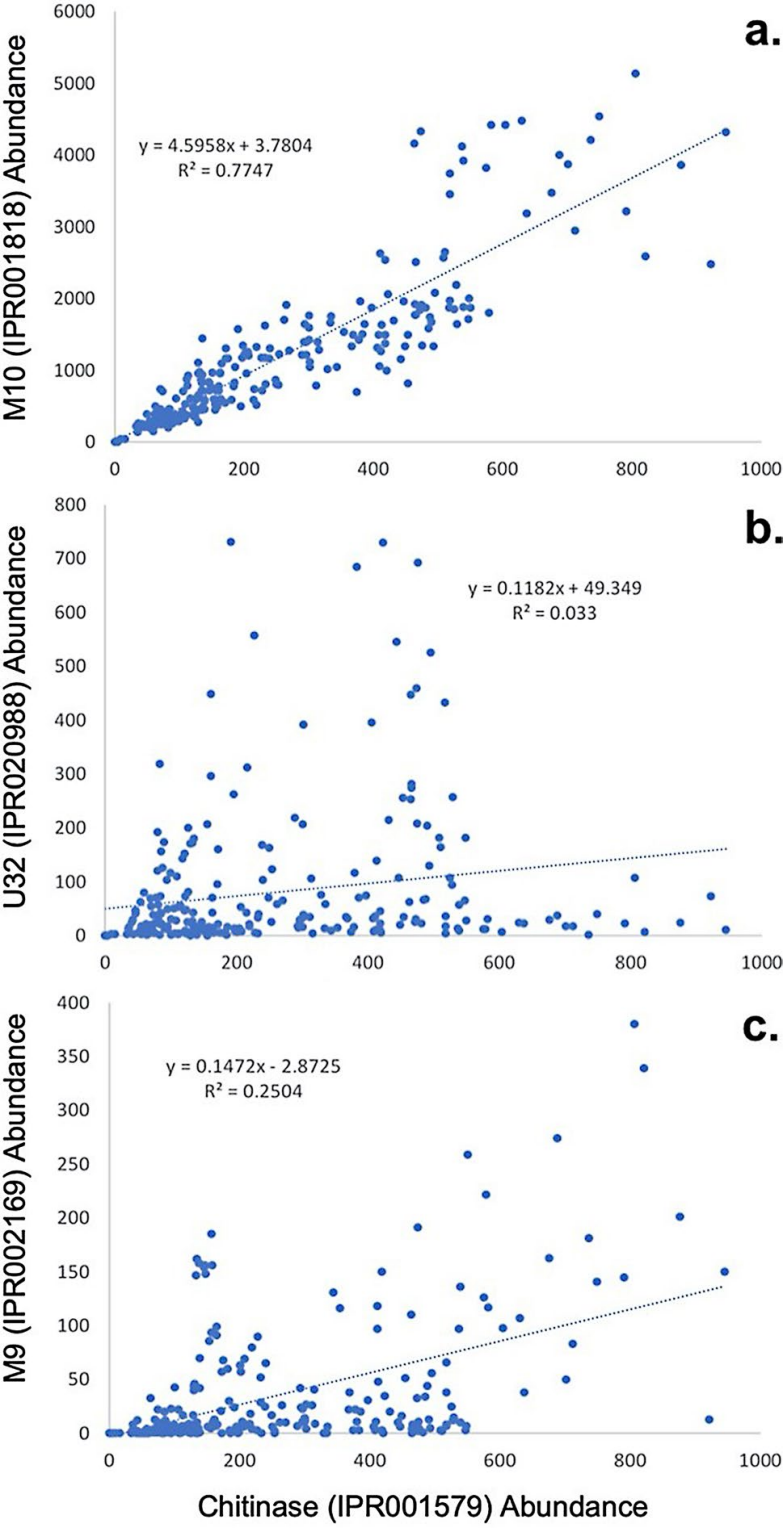
Metagenomic co-occurrences of chitinases with MMPs (**a**), U32 collagenases (**b**), and M9 collagenases (**c**). Each point represents an individual metagenome. Abundances of genes are based on the number of inferred protein sequences in a given metagenome that map to a particular gene. Ordinary least squares (OLS) regressions and associated R^2^ values are reported

Based on the hypothesis that bacteria use MMPs to degrade animal-derived collagen, abundances of reads encoding MMPs and chitinases should be positively correlated, as chitinases represent another family of enzymes that target animal-derived substrates available in the marine environment. Indeed, across all 248 samples, these two genes frequently co-occur (R^2^ = 0.683, *p* < 10^− 5^) and the correlation can be further improved (R^2^ = 0.775, *p* < 10^− 5^) with the removal of a single outlier (TARA_B100001059) sampled from a deep chlorophyll maximum off the coast of Chile with an overabundance of chitinase sequences (Fig. 2a). Abundances of M9 microbial collagenases were only weakly correlated with chitinase and MMP abundances (R^2^ = 0.2504, *p* < 10^− 5^; R^2^ = 0.194, *p* < 10^− 5^) (Fig. 2c), while U32 collagenase abundances were correlated with neither (R^2^ = 0.033, *p* = 0.39; R^2^ = 0.0082, *p* = 0.155**) (**Fig. 2b). Therefore, MMPs are not only implied to be directly involved with the utilization of animal collagen, but their presence in microbial genomes appears to be a specific adaptation to do so, as this strong correlation is not even observed for other bacterial enzymes that have been experimentally confirmed to cleave collagen. That being said, bacterial or archaeal MMP’s ability to cleave animal collagen has yet to be investigated experimentally, which would provide the strongest support for this evolutionary scenario.

### Phylogeny and taxonomic groupings of MMP sequences

**Fig. 3:**
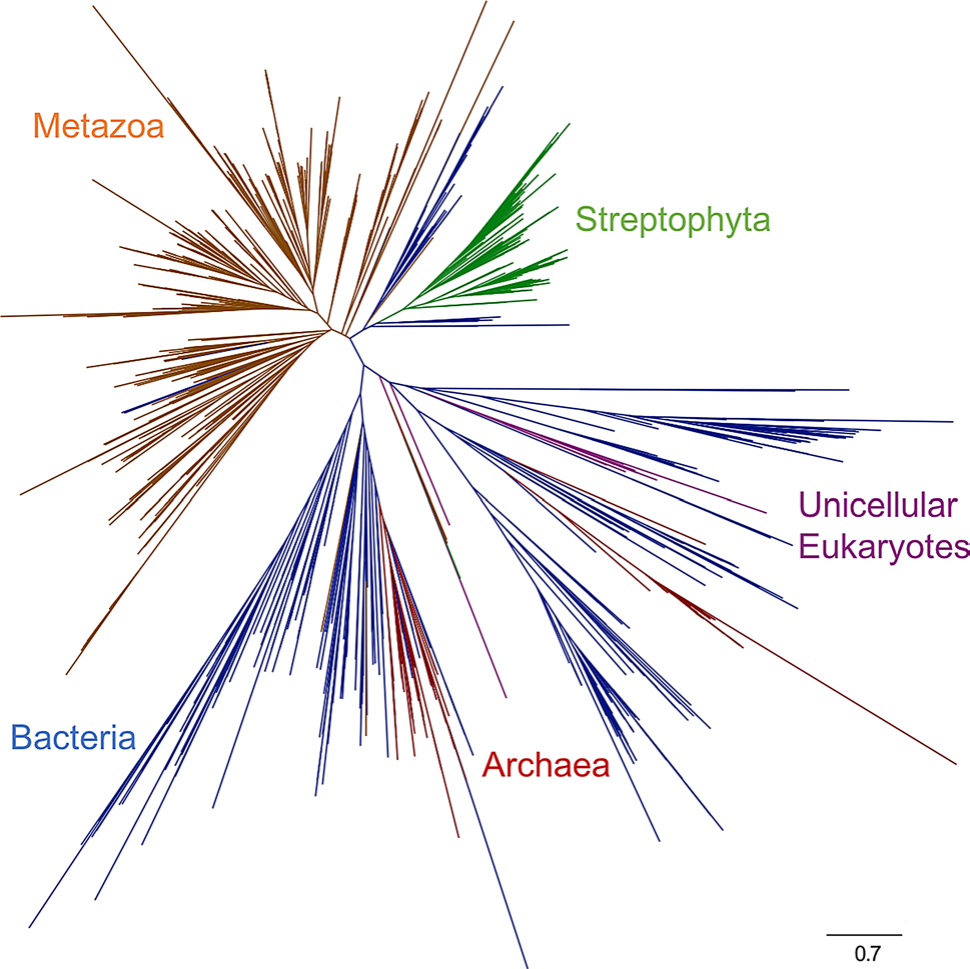
Unrooted maximum likelihood phylogeny of MMP sequences. Colors correspond to the labeled major taxonomic groupings

The unrooted MMP phylogeny contains three major taxonomic groupings: (1) Bacteria and Archaea, (2) Metazoa, and (3) Streptophyta (Fig. 3). As the taxonomic distribution of MMPs across the tree of life is shallow and sparse, and as there has been so little sequence divergence between eukaryotes and bacteria, a vertical-inheritance interpretation can be ruled out; it is clear that the distribution of MMPs across the tree of life is a product of HGT, with multiple events involving Metazoa, Bacteria, Archaea, and Streptophyta. The rooting of the tree and inferred directionality of the HGT events is therefore of crucial importance in reconstructing the evolutionary history of this protein family

### Metazoan MMP sequences

Despite metazoan monophyly, the MMP phylogeny does not recover expected relationships between descendant phyla, and distantly related phyla are often sister to one another within the tree. However, within-phylum diversity is generally much more consistent with the species trees for those particular groups (e.g., in vertebrate subtrees, actinopterygian fish usually diverge before tetrapods). Due the rarity of HGT between metazoans [41, 42], HGT is not a favored hypothesis to explain this history. Most deep branches are very short with low support values, indicating rapid gene duplication and diversification of metazoan MMPs. As the earliest speciation event in this portion of the tree should be the divergence of Cnidaria from Bilateria, the number of different clades of cnidarian MMPs provides a lower bound for the number of times the MMP gene was duplicated prior to cnidarian divergence, which informs the number of copies present in the last common ancestor of all eumetazoans. From this reasoning, based on the number of groups of cnidarian MMPs, the last common ancestor of Eumetazoa had at least 8 distinct MMP copies.

One striking feature of this phylogeny is the fact that vertebrate diversity is mostly confined to two large clades. Those large clades contain evidence for extensive duplication within Vertebrata following their crown group diversification, possibly coinciding with loss of most ancestral MMPs. Such a hypothesis is further supported by the scattered representation of invertebrate chordate (Cephalochordata and Urochordata) MMPs throughout the tree. The metazoan sequences that group closest to microbial and embryophyte sequences are a well-supported group of nematodes and arthropods (95.1/96 SH-aLRT/UFBoot). However, while the clade itself is well supported, its placement within the tree is less so (86.8/60 SH-aLRT/UFBoot), possibly due to wandering sequences, including one group of vertebrate MMPs on a long branch.

### Bacterial and archaeal MMP sequences

MMPs are widely distributed throughout the bacterial domain, with Pfam searches recovering representatives in at least 1,039 different species [43]. It is difficult to identify clear phylogenetic signals within bacterial MMP sequences, due to short, poorly supported internal branches, but closely related groups do tend to cluster together. Approximately 15% of the 1677 bacterial MMP sequences in Pfam’s database belong to *Lactobacillus*, with representatives in 31 different species. One large well-supported (98.7/98 SH-aLRT/UFBoot) clade in our tree is comprised of various *Lactobacillus* and *Streptococcus* species, with *Streptococcus* nested within *Lactobacillus*, indicating an HGT from *Lactobacillus*.

Another well supported group (98.5/99 SH-aLRT/UFBoot) contains representatives of Candidate Phyla Radiation (CPR) group and some proteobacteria which appear to be recipients of HGTs from within the CPR. Parcubacteria appear to have been the first clade of CPR to obtain MMPs, later transferring it to Microgenomates, as the only representatives of the Microgenomates are in a small group (96.4/100 SH-aLRT/UFBoot) nested within the Parcubacteria.

**Fig. 4:**
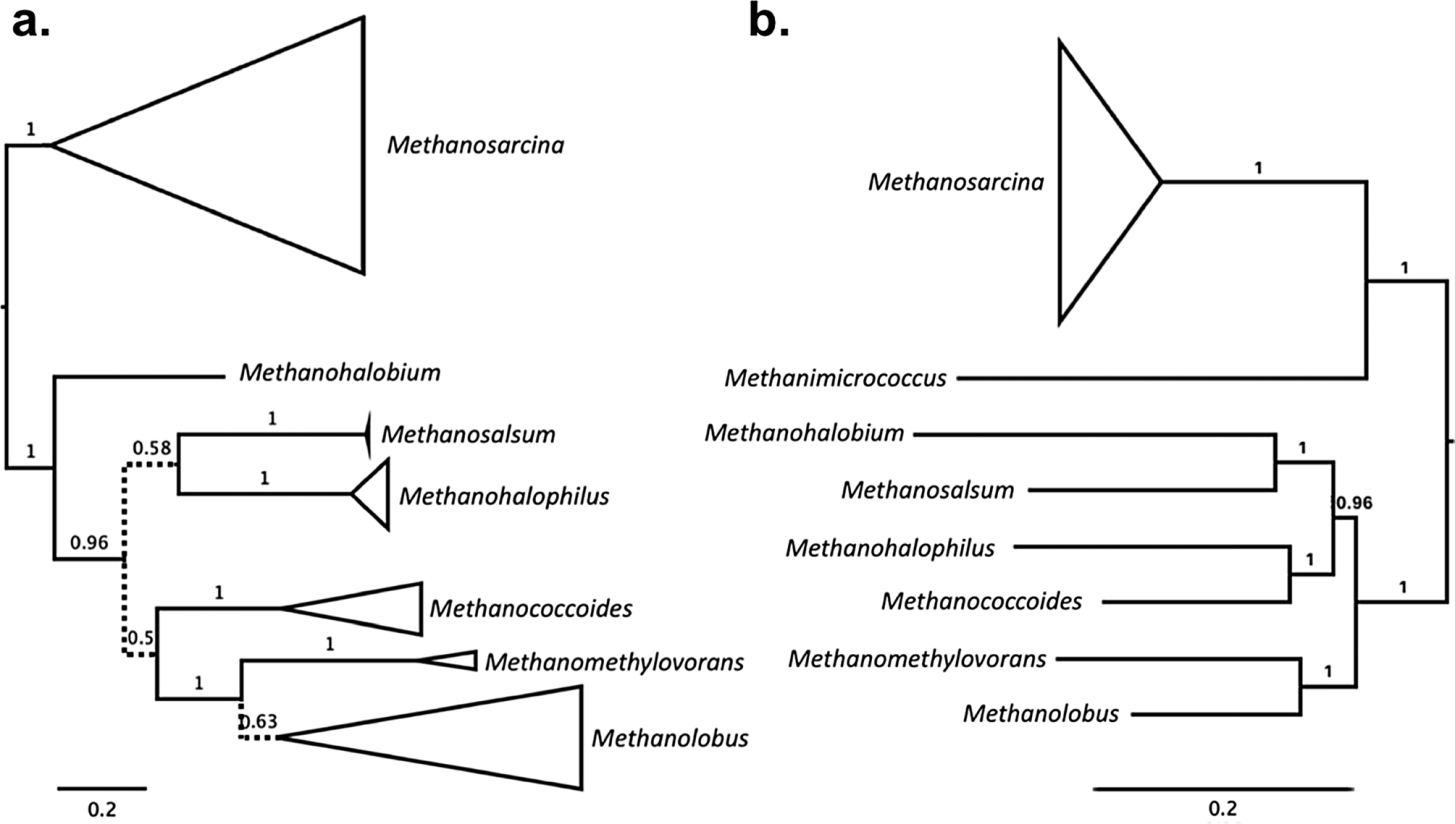
Comparison between MMP phylogeny (**a**) and Methanosarcinaceae species tree (**b**). Both trees are posterior consensus trees generated using Bayesian inference. Nodes are collapsed to the genus level and node width corresponds to number of sequences. Posterior probabilities are labelled on internal nodes. Dotted branches denote poorly supported bipartitions. The MMP phylogeny is manually rooted to be congruent with the species tree

The few archaeal MMP sequences within the phylogeny rarely group with one another, implying that most archaeal MMPs have been acquired by relatively recent HGT. The largest cluster of archaeal sequences consists of a well-supported (90.3/97 SH-aLRT/UFBoot) group of methanogens belonging to family Methanosarcinaceae, which does appear to be mostly congruent with the expected species tree history for this group (Fig. 4). This suggests that divergence times within Methanosarcinaceae may be informed by this HGT event, by constraining this clade to be younger than the origin of collagen within animals (i.e., younger than crown animal diversification). Some additional Methanosarcinaceae sequences do not group with this large clade, indicating that there have also been some more recent gene transfers into members of this clade. Other represented Archaea include members of Nitrospumilales, Halobacteriales, and Pacearchaeota, but no useful species-tree structure is observed for these groups, as they are only represented by very sparse taxonomic sampling.

### Streptophyte sequences

**Fig. 5:**
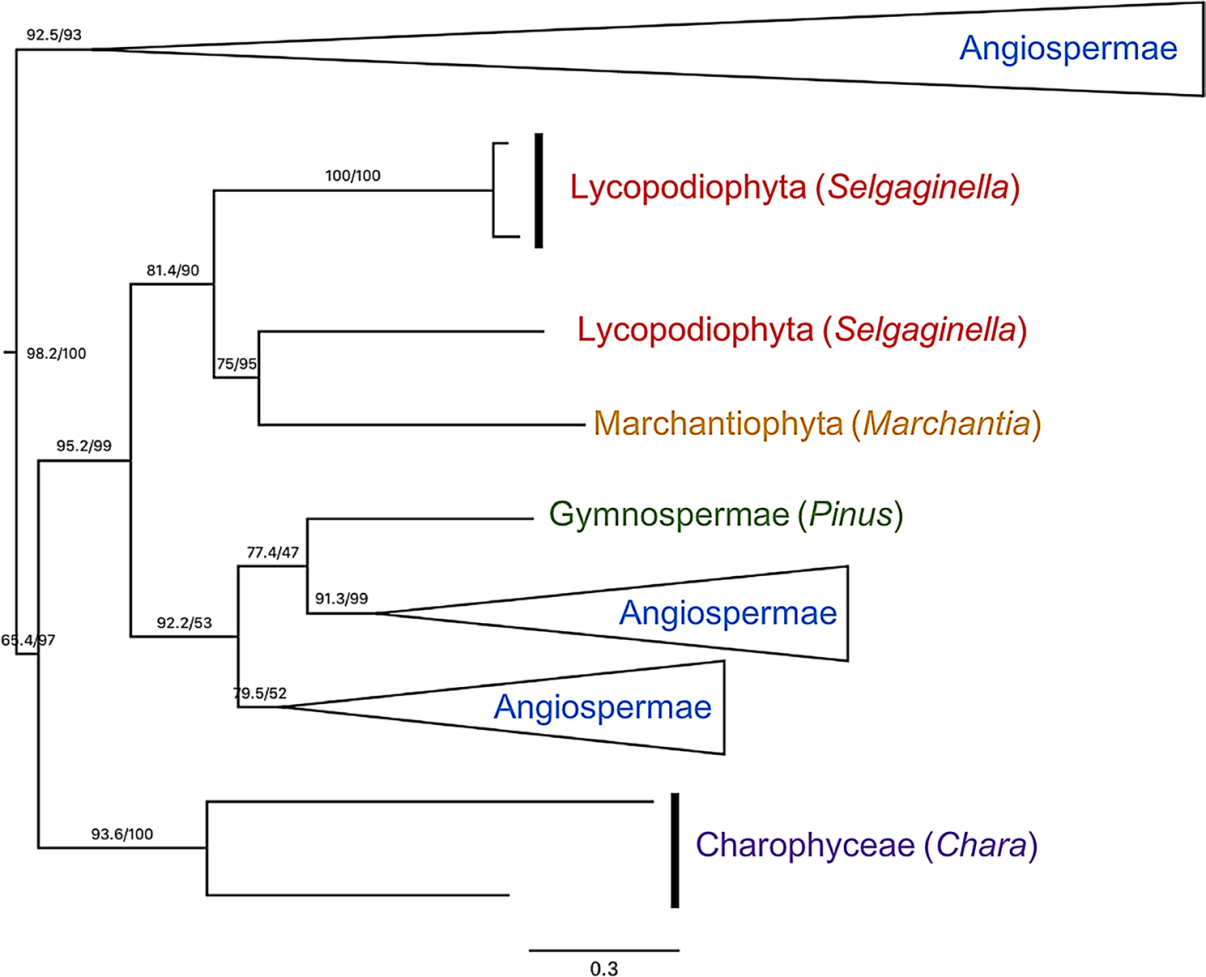
Subtree of MMP phylogeny to which almost all plant diversity is localized. Groups are colored based on major embryophyte divisions. Values on branches indicate Shimodaira–Hasegawa approximate likelihood ratio test/ultrafast bootstrap (SH-aLRT/UFBoot) values

The diversity of plant MMPs is dominated by angiosperms, with only a few representatives from other streptophytes. All of these sequences, aside from a small clade of chlorophytes, fall within one well-supported monophyletic group (98.2/100 SH-aLRT/UFBoot), which contains two additional subgroups (Fig. 5). The first of the two subgroups is well supported (92.5/98 SH-aLRT/UFBoot), and only contains sequences belonging to eudicots. As this subtree is not rooted on the basal eudicots (Ranunculales and Proteales) this group is either mis-rooted or has a history complicated by duplications and losses. The number and distribution of rosid and asterid sequences indicates that there were at least 4 duplication events prior to the crown divergence of Pentapetalae. The other subtree is also well-supported (95.4/97 SH-aLRT/UFBoot) and is far more congruent with the plant species tree, with Charophyceae as the outgroup to Embryophyta, Lycophyta and Marchantiophyta as basal embryophytes (although the latter is incorrectly nested within the former), and Spermatophyta (gymnosperms) sister to Magnoliophyta (angiosperms). Most of this clade’s diversity is still represented only by eudicots, but the few available basal embryophyte MMP sequences make it clear that this subtree contains the original split between Charophyceae and Embryophyta, indicating that these plants received an MMP gene via HGT prior to that divergence. However, it is difficult to explain why no basal spermatophytes are represented in the first subtree. This may either indicate an incorrect phylogenetic reconstruction, or a number of gene loss events within this group.

### Other eukaryal MMP sequences

In addition to animals and plants, some other eukaryotes appear to have MMPs. Most notably, one set of eukaryotes deeply nested within bacterial diversity consists largely of distantly related unicellular photosynthetic eukaryotes, including haptophytes, green algae, and Pelagophyceae, in addition to a heterotrophic group of heterokonts, Labyrinthulomycetes. While the overall grouping is poorly supported (56.7/51 SH-aLRT/UFBoot), the larger of two constituent subgroups is fairly well supported (89.8/100 SH-aLRT/UFBoot). These taxa tend to occupy similar environments, suggesting HGT between groups as an explanation for their phyletic clustering. Alternatively, the large diversity within this group of eukaryotic sequences may indicate an unsampled diversity of protist MMPs. Additionally, fungal species are scattered throughout the bacteria within the MMP phylogeny, represented by single taxa in each instance.

### Rooting

Determining the root of the MMP phylogeny allows for the inference of both the origin of MMPs with respect to animal evolution, as well as the directionality of any observed HGT events that inform the co-evolution of animals and microbial metabolisms. A root within Metazoa would be consistent with this protein family originating as part of animal physiology, used in the breaking down of tissues during growth and developmental processes. The presence of MMPs in other lineages, including microbes, would then indicate subsequent HGTs into those groups. Alternatively, a root outside of Metazoa, for example, within Bacteria, would suggest that MMPs originated in response to the presence of animal proteins within the environment, and were subsequently acquired via HGT by animals themselves.

The most reliable method for rooting a phylogeny is to identify and include sequences representing an outgroup. The bipartition separating the outgroup from the ingroup in the resulting larger tree will therefore, if reliably reconstructed, identify the root of the ingroup. Protein structural analyses suggest that MEROPS family M12, which contains the largely metazoan groups M12A and M12B, the astacins and adamalysins, are the most closely related proteins to MMPs, and can therefore potentially serve as an outgroup for rooting. Structural alignments between M12B peptidases and MMPs demonstrate well-conserved secondary structure between the two groups, which indicates shared ancestry [44]. However, at the level of individual amino acids, sequence alignment between M12B and MMP family proteins reveals substantial divergence between the two groups, having only ~ 25% sequence similarity. Accordingly, generated phylogenies show a very long branch separating the two groups.

**Fig. 6:**
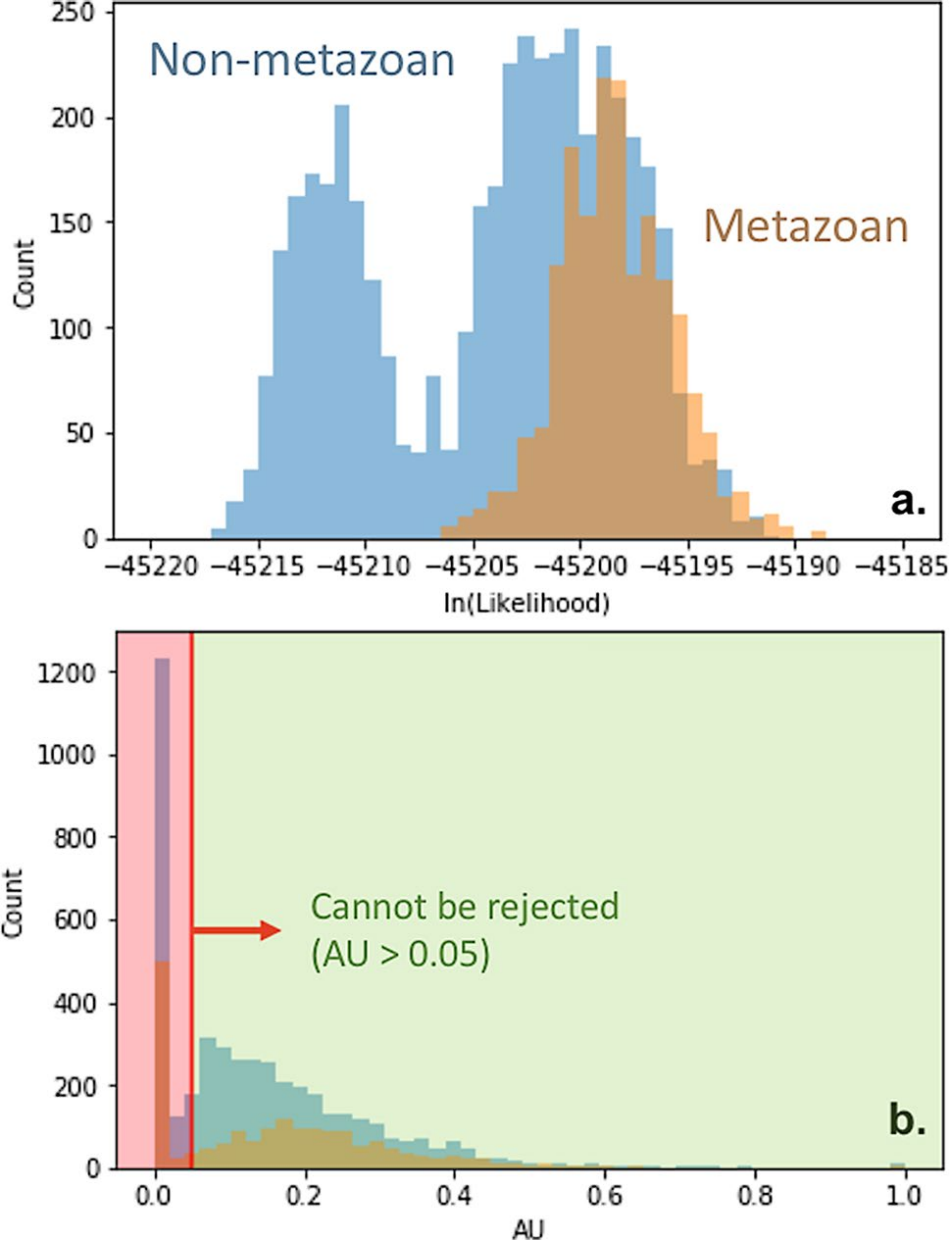
Likelihood analysis of alternative outgroup rootings for the MMP phylogeny. **a**. Histogram of likelihoods of all alternatively rooted trees. Possible metazoan roots (orange) are overlain with possible non-metazoan roots (blue). **b**. Histogram of approximately unbiased likelihood test *p*-values of all alternatively rooted trees. The AU test fails to reject 69.5% of all animal-rooted trees and 68.3% of all Bacteria-rooted trees

Although the maximum-likelihood tree including this outgroup produced a metazoan rooting for MMP, closer inspection reveals this to be a tip rooting on an especially derived echinoderm sequence, suggestive of a long branch attraction artefact. Likelihood analysis of a total subtree prune and re-graft (SPR) operation revealed that this outgroup rooting cannot be used to distinguish between a bacterial and metazoan root, as placements of M12B within Bacteria and within Metazoa yield statistically indistinguishable likelihood values (Fig. 6a). The AU test [45] was unable to reject 69.5% of Metazoan-rooted trees and 68.3% of bacterially-rooted trees generated (Fig. 6b).

### Novel MMP-derived age constraints

Independently of the topology of the donor-recipient tree, HGTs of MMP genes can still be used to impose older bound age constraints on recipient clades. As this gene would presumably only be acquired by groups degrading animal collagen, such acquisitions and the associated recipient clades must postdate crown Metazoa. Despite some uncertainty in the exact age of crown Metazoa [27], a conservative older bound estimate of ~ 850 Ma can still be highly informative when applied to otherwise unconstrained microbial divergences.

Using this logic, older bounds for the ages of two non-metazoan groups can be derived from the MMP phylogeny. The first, the age of the embryophyte-charophyte divergence, represents an internal test of the validity of MMP-derived age constraints, as this age is already well-established by fossil and molecular clock evidence. A recent fossil-calibrated molecular clock study placed the age of this divergence between 750 and 550 Ma, with a mean age of 635 Ma [46]. This divergence substantially postdates animal origins, and therefore is compatible with the crown Metazoa MMP-derived age constraint, as well as a more stringent constraint using older-bound age estimates for the divergence between Cnidaria and Bilateria at 636.1 Ma [47], if the HGT donor to plants diverged after this split, as is observed in our tree topology.

The other constraint, the age of crown Methanosarcinaceae, is potentially highly informative, as few, if any, older-bound age constraints exist that can be readily applied to archaeal molecular clock analyses. Previously, Methanosarcinaceae has been calculated to have diversified between 2.1 and 0.75 Ga, with a mean age of 1.53 Ga [23], although this age estimate relied on a single distant younger-bound calibration deeper within the tree, rather than active calibrations within Methanosarcinales.

### Revised molecular clock analysis of Methanosarcinaceae

**Fig. 7:**
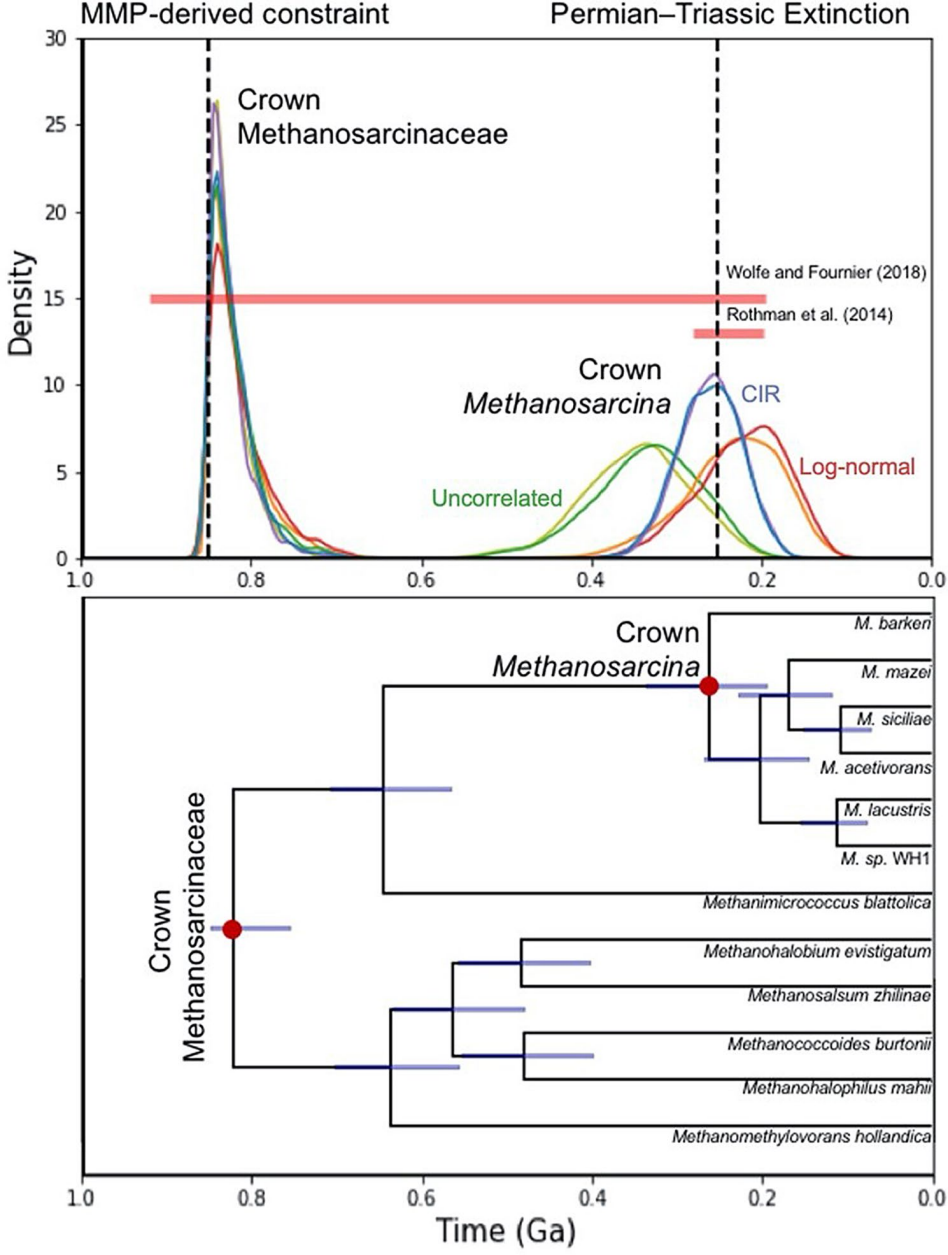
Posterior age estimates for the divergence of crown Methanosarcinaceae and *Methanosarcina*. The upper panel shows the posterior density distributions of ages of the two nodes of interest under different models. The horizontal red bars indicate the estimated age of crown *Methanosarcina* from previous studies. The left vertical dotted line indicates the age constraint (< 850 Ma) imposed on crown Methanosarcinaceae and the right one shows the approximate timing of the End Permian extinction event. Distributions are colored by branch rate model and prior. Red: log-normal/birth-death, Orange: log-normal/uniform, Blue: CIR/birth-death, Purple: CIR/uniform, Green: uncorrelated/birth-death, Olive: uncorrelated/uniform. The lower panel shows the relevant subtree of the chronogram generated by the CIR/birth-death model, with 95% confidence intervals marked on each internal node.

Revising the molecular clock analysis of Wolfe and Fournier (2018) with the addition of this calibration does indeed substantially decrease the age estimate for crown Methanosarcinaceae, producing a mean age estimate of 0.83 Ga (0.849 − 0.755 Ga), under a CIR process with a uniform prior. Evolutionary rate model choice did not substantially impact this result, as would be expected given that this node’s age is mostly being influenced by the calibration (Fig. 7). The resulting age of *Methanosarcina*, a genus within Methanosarcinaceae, is of particular interest, as the crown age of this group has been previously estimated to be 240 ± 41 Ma, thereby implicating *Methanosarcina* in the carbon cycle breakdown associated with the Permian-Triassic Extinction (PTE) at around 252 Ma [48]. This age estimate was obtained by using an older-bound constraint on Methanosarcina, imposed by the HGT of acetoclastic genes from a group of cellulose-degrading bacteria [24]. The revised mean age for crown *Methanosarcina* aligns remarkably well with the PTE at 251.9 Ma [49] for CIR-process molecular clock models under both birth-death and uniform priors, with highest probability density (HPD) ages of 261.9 Ma and 259.4 Ma, respectively (Fig. 7). However, this age estimate does vary with the evolutionary rate model, with log-normal distribution models producing slightly younger mean age estimates (221.9 Ma and 233.4 Ma, respectively) and uncorrelated gamma distribution models producing older estimates (334.7 Ma and 346.2 Ma, respectively). Under all models, the PT extinction is solidly within the bounds of the posterior probably density. By independently reaching the same divergence time estimate using more proximal calibration data, our result supports both the conclusions of Rothman et al. (2014) and the validity of this age constraint.

## Discussion

### In which taxa did MMPs originate?

**Fig. 8:**
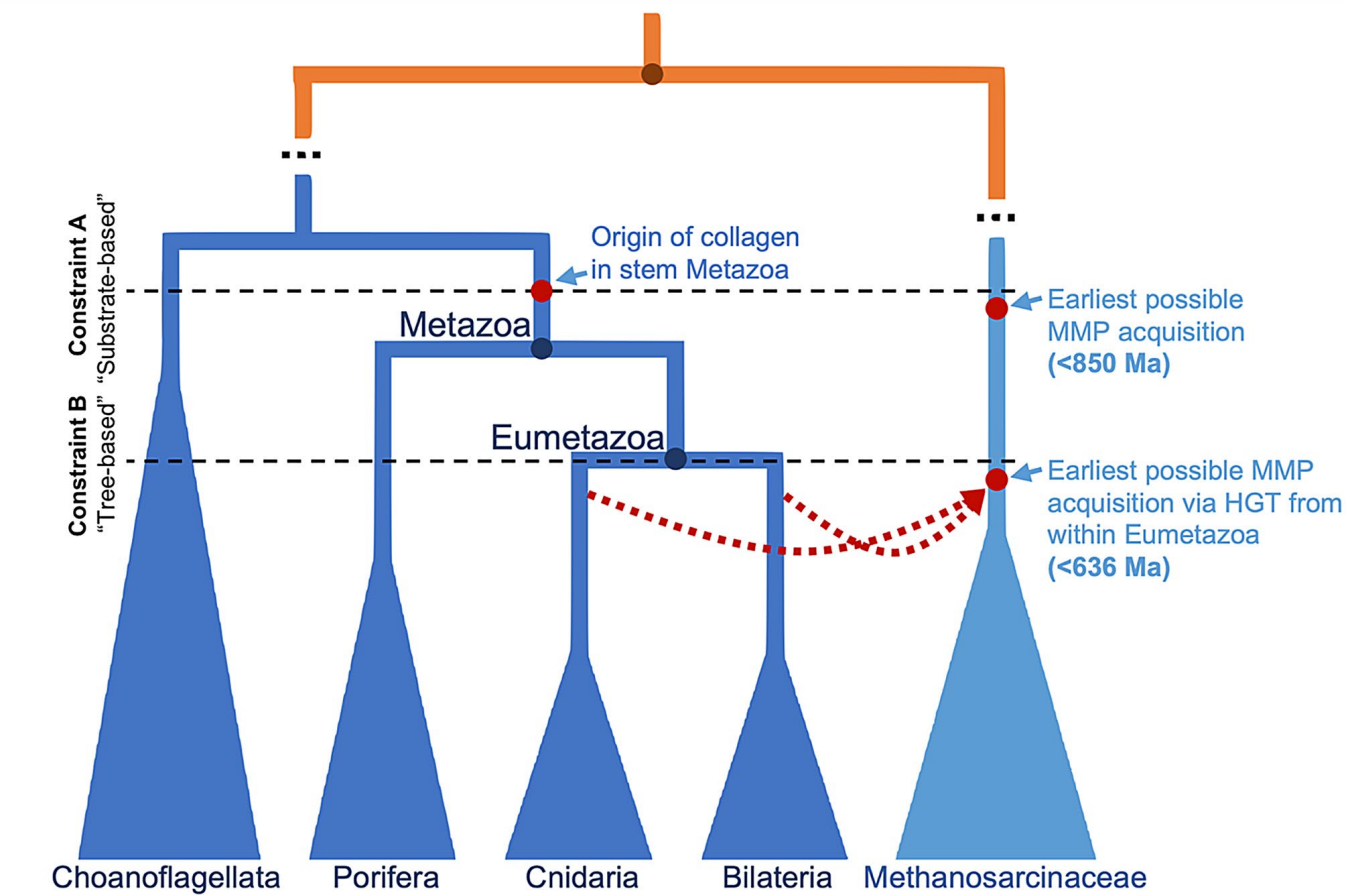
Evolutionary history of MMPs and collagen in Metazoa and Methanosarcinaceae. The timing of the crown divergence of Methanosarcinaceae can be constrained by the timing of their MMP acquisition, which itself can be constrained by **(A)** the origin of collagen, the primary MMP substrate, or, more speculatively, by **(B)** the topology of the MMP phylogeny.

The directionality of any HGT events within the MMP phylogeny can only be inferred topologically if the location of the root of the tree is sufficiently constrained. Based on the unrooted topology, there are three hypothetical primary HGT scenarios, each associated with a distinct rooting scenario: (1) Bacteria-to-Metazoa and Bacteria-to-Plantae independently, (2) Metazoa-to-Bacteria-to-Plantae, and (3) Plantae-to-Bacteria-to-Metazoa. Distinguishing between these three scenarios requires inference of the root of the tree, with a bacterial, metazoan, and plant root supporting scenarios 1, 2, and 3, respectively (Fig. 8). Based on the topology of the tree, the only clear conclusion regarding rooting is that, since Plantae’s subtree appears to be correctly rooted, the root does not lie within Plantae, ruling out scenario 3.

The MMP phylogeny highlights the challenges of rooting gene trees that involve multiple transfers between eukaryal and microbial groups, especially in the presence of gene duplication events. One characteristic of the MMP phylogeny that makes rooting difficult is that the animals and plants tend to be on much shorter branches than the bacterial groups. A rooting method like minimal ancestor deviation [50] or midpoint rooting [51], which are based on tree branch lengths, are therefore especially likely to place the root within the prokaryotic diversity, as such a root will reduce the inferred deviation from clocklike evolutionary rates. However, there is no reason to expect consistent substitution rates across the MMP phylogeny, due to the wide taxonomic diversity and the large number of internal branches which presumably contain HGT events, and are therefore likely to be especially long. Gene-species tree reconciliation methods are also unsuitable to infer the correct root for a tree like this, as such methods rely on accurate tree topology, which the MMP tree lacks due to short branches and uncertain deep relationships caused by the short alignment. Furthermore, expected frequencies for duplication, loss, and transfer vary across the Tree of Life, with fixed gene duplications being more common within Eukaryotes (especially Metazoans), and HGTs being more common within microbes [52, 53]. Since there is currently no software that supports weighting the penalties for different reticulation events between particular clades, transfer events between animals would be scored as equally plausible as transfer events between bacteria, and possibly erroneously favored over duplication-loss scenarios. Additionally, recent work has suggested that gene-species tree reconciliation may be ineffective at root inference when there are substantial departures from the species tree [54].

Nevertheless, a few clues do exist regarding the root. For one, the extensive bacterial diversity of MMPs would seem to imply a bacterial root, as a metazoan root would give only around 850 million years for this gene to have spread throughout the kingdom. Secondly, the existence of many bacterial copies of the distant outgroup, M12, may suggest that both of these genes were acquired by eukaryotes due to transfers from bacterial diversity, albeit with a substantially earlier transfer for M12 than the MMPs. This hypothesis may warrant a follow-up study of the M12 phylogeny. In contrast, however, recent work on one family of chitinases demonstrates that they initially arose in chitin-producing fungi, and were later transferred into multiple bacterial clades, presumably in response to increased chitin availability [25].

With either a metazoan or bacterial root, the following two evolutionary events presumably occurred: (a) early animals evolved (or horizontally acquired) MMP genes alongside ECM components like collagen, and (b) bacteria evolved (or horizontally acquired) MMP genes, in order to take advantage of new animal metabolites. The subsequent rise in total animal biomass would have increased not only collagen availability, but also the number of opportunities for gene transfer between microbes and metazoans.

### Do bacterial MMPs degrade animal matrix proteins?

Due to the lack of experimental testing of bacterial MMP function, it is currently unknown whether or not bacterial MMPs catalyze the same reactions as animal MMPs, namely the cleavage of animal ECM proteins. The most obvious argument to be made in favor of consistent function across MMP diversity is the relatively high sequence homology between the two groups, which is how the bacterial MMPs were identified initially. The high co-occurrence of bacterial MMPs with chitinases in marine metagenomes lends further support to the hypothesis that bacterial MMPs cleave animal ECM proteins, as chitinases would only be expected to appear in the presence of chitin-producing organisms, which, in the context of marine ecosystems, are likely to consist mostly of animals (arthropods). Additionally, the fact that other bacterial collagenases lack this correlation may be due to their lack of substrate specificity, suggesting their use in roles other than ECM protein degradation and implying higher substrate specificity for bacterial MMPs. Once again, however, this high correlation is not conclusive and does not preclude the possibility that bacterial MMPs can degrade collagen, but are also used to degrade other molecules not associated with animals. Furthermore, based on protein-mapping alone, the extent to which these genes co-occur within individual genomes is ambiguous, so it is possible that this correlation is a product of certain environments favoring carbon-degrading generalists, rather than collagen- or chitin-degrading specialists. Still, even intragenomic correlation would imply similar functionality between the two groups of enzymes.

## Conclusions

This study represents further leveraging of the genomic record to supplement the poor microbial fossil record. To this end, a crucial age constraint was revealed within Archaea, a microbial group with an extremely limited fossil record and largely unconstrained ages. Phylogenetic analysis of MMPs yielded an upper bound of 850 Ma for divergence of Methanosarcinaceae, a clade of methanogens previously inferred to be substantially older. Additionally, this study highlights a rare mechanism for animal-to-plant horizontal gene transfer in which intermediate bacterial diversity carries a gene between the two groups. Future studies should attempt to determine the effects of this new constraint on molecular clock-based methanogen age estimates, as well as experimentally confirm the collagenolytic activity of MMPs found within Methanosarcinaceae.

## Supplementary Information

Below is the link to the electronic supplementary material.

Supplementary Material 1

Supplementary Material 2

Supplementary Material 3

Supplementary Material 4

Supplementary Material 5

Supplementary Material 6

Supplementary Material 7

Supplementary Material 8

Supplementary Material 9

Supplementary Material 10

Supplementary Material 11

Supplementary Material 12

## Data Availability

The datasets supporting the conclusions of this article are included within the article (and its additional files).
