# Supplementary material for: Horizontal transfer of matrix metalloproteinase genes links early animal and microbial evolution
Source: Biol Direct. 2025 Nov 5;20:107. doi: 10.1186/s13062-025-00700-4 (PMC12587646; doi:10.1186/s13062-025-00700-4)
Supplement: Supplementary file 1 — Supplementary Material 1 [file 13062_2025_700_MOESM1_ESM.docx]

**Description of Supplemental Files**

All trees are in Newick format. Alignments used with IQTree are in FASTA format (.afa), while those used for Phylobayes runs are in PHYLIP format (.phy). Sequences are labeled with accession numbers followed by taxonomic information. There is a mix of NCBI and UniProtKB accession IDs.

1. MMP_full.tree, MMP_full.afa

Complete MMP phylogeny, and the sequences used to generate it. Seen in Figure 3. Figure 5 is a subtree of this phylogeny.

2. methanosarcinaceae_MMP.tree, methanosarcinaceae_MMP.phy

Bayesian tree of MMP sequences from Methanosarcinaceae, and the sequences used to generate it. Seen in Figure 4a.

3. methanosarcinaceae_ribosomal.tree, methanosarcinaceae_ribosomal.phy

Methanosarcinaceae species tree constructed from a concatenated ribosomal protein alignment, as well as the alignment itself. Seen in Figure 4b. Informed the topology of ribosomal_chronogram.tree.

4. ribosomal_chronogram.tree, ribosomal_chronogram.phy

Ribosomal protein alignment from Wolfe and Fournier (2019) supplemented with additional taxa and an 850 Ma constraint applied to crown Methanosarcinaceae to generate a new chronogram. This chronogram was generated using a CIR process and a birth-death prior. Seen in Figure 7.

5. M10M12_outgroup.tree, M10M12_outgroup.afa

Attempt to root the MMP phylogeny using M12B as an outgroup. M12B ultimately proved too evolutionarily distant for reliable root inference.
